# Energy Landscape of Alginate-Epimerase Interactions Assessed by Optical Tweezers and Atomic Force Microscopy

**DOI:** 10.1371/journal.pone.0141237

**Published:** 2015-10-23

**Authors:** Armend Gazmeno Håti, Finn Lillelund Aachmann, Bjørn Torger Stokke, Gudmund Skjåk-Bræk, Marit Sletmoen

**Affiliations:** 1 Biophysics and Medical Technology, Dept. of Physics, Norwegian University of Science and Technology, NO-7491, Trondheim, Norway; 2 NOBIPOL, Dept. of Biotechnology, Norwegian University of Science and Technology, NO-7491, Trondheim, Norway; LAAS-CNRS, FRANCE

## Abstract

Mannuronan C-5 epimerases are a family of enzymes that catalyze epimerization of alginates at the polymer level. This group of enzymes thus enables the tailor-making of various alginate residue sequences to attain various functional properties, e.g. viscosity, gelation and ion binding. Here, the interactions between epimerases AlgE4 and AlgE6 and alginate substrates as well as epimerization products were determined. The interactions of the various epimerase–polysaccharide pairs were determined over an extended range of force loading rates by the combined use of optical tweezers and atomic force microscopy. When studying systems that in nature are not subjected to external forces the access to observations obtained at low loading rates, as provided by optical tweezers, is a great advantage since the low loading rate region for these systems reflect the properties of the rate limiting energy barrier. The AlgE epimerases have a modular structure comprising both A and R modules, and the role of each of these modules in the epimerization process were examined through studies of the A- module of AlgE6, AlgE6A. Dynamic strength spectra obtained through combination of atomic force microscopy and the optical tweezers revealed the existence of two energy barriers in the alginate-epimerase complexes, of which one was not revealed in previous AFM based studies of these complexes. Furthermore, based on these spectra estimates of the locations of energy transition states (*x*_*β*_), lifetimes in the absence of external perturbation (*τ* ^*0*^) and free energies (*ΔG* ^#^) were determined for the different epimerase–alginate complexes. This is the first determination of *ΔG* ^#^ for these complexes. The values determined were up to 8 k_B_T for the outer barrier, and smaller values for the inner barriers. The size of the free energies determined are consistent with the interpretation that the enzyme and substrate are thus not tightly locked at all times but are able to relocate. Together with the observed different affinities determined for AlgE4-polymannuronic acid (poly-M) and AlgE4-polyalternating alginate (poly-MG) macromolecular pairs these data give important contribution to the growing understanding of the mechanisms underlying the processive mode of these enzymes.

## Introduction

Alginate, a polysaccharide derived from brown algae and the bacterial genera *Pseudomonas* and *Azotobacter*, is a versatile biopolymer by virtue of its biocompatibility and ability to form calcium-induced ionotropic hydrogels compatible with living cells. Such properties have paved the way for the use of alginates within various biomedical fields, such as drug delivery, regenerative medicine and cell encapsulation for cell transplantation [1–5]. Alginate is initially synthesized as a mannuronan homopolymer (poly-M) followed by maturation towards the final alginate residue sequence in an epimerization process. This epimerization occurrs at the polymer level and are catalyzed by C-5 epimerases. The alginate producing bacteria *Azotobacter vinelandii* express a family of isoenzymes AlgE 1–7 that catalyze the epimerization of β- d-mannuronic acid (M) residues within the alginate chains to their epimer: the α-l-guluronic acid (G) residues. The sequence of the introduced α-l-Gul*p*A is specific for the particular epimerase [6, 7]. The introduction of G residues alters the chain stiffness [8] and affinity for divalent ions (e.g. Ca^2+^, Ba^2+^, Sr^2+^) [9], which allows alginate chains with sufficiently long G-sequences to form physical cross-links with divalent metal cations and form hydrogels. Alginates with polyalternating sequences (poly-MG) have also been shown to form ionotropic hydrogels [2, 10, 11], however, these are much weaker than those made from alginates with a high content of G-blocks.

**Fig 1:**
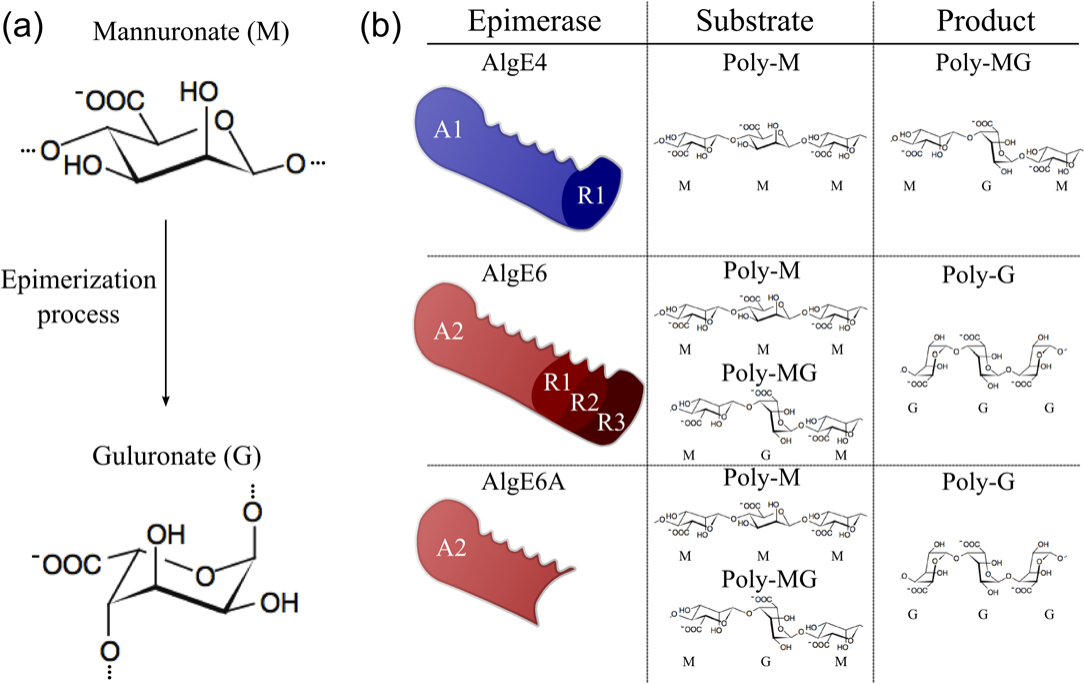
The epimerization process, epimerases and prevailing alginate residue sequences of the various epimerase substrates and resulting products. *(a)* The mannuronan C-5 epimerases possess the ability to epimerize β-d-mannuronate residues (M) to its epimer form; α-l-guluronate residue (G). *(b)* The naturally occurring epimerases are known to form long stretches of systematically epimerized alginates. While AlgE4 can produce polyalternating structures from polymannuronic alginates, AlgE6 can epimerize both polymannuronic and polyalternating structures to form polyguluronic alginates.

The AlgE mannuronan C-5 epimerases are known to produce highly ordered alginates with long repeating units of GG or MG blocks from pure poly-M alginates [6, 7] (Fig 1). AlgE6, for instance, has been found to epimerize poly-M through initial production of poly-MG and subsequent reprocessing of the polyalternating alginate chains to form alginates with long stretches of G-residues [12, 13]. In contrast, AlgE4 ends the epimerization process after poly-MG has been formed from poly-M [12, 14, 15]. This difference in enzymatic action is associated with enzyme structure [16]. All AlgE epimerases consist of two types of structural modules, designated A (~385 amino acids each, with 1 or 2 copies) and R (~155 amino acids each, with one to seven copies) [6]. The catalytic site is known to be located at the A-modules, and the R-modules are thought to modulate the epimerization rate by stabilizing the process with an extended sub-site binding the alginates. The R-modules were recently shown to also play a role in the epimerization pattern of the final alginate product [13]. Indeed, A-modules are known to epimerize alginates autonomously [16], however the action of A-modules is dependent on the presence of R-modules [16]. The different A-modules share ~85% primary amino acid sequence homology. Despite of this large sequence homology, the epimerases catalyze residue sequence in the alginate product that strongly depends on the epimerase used. Recently, it has been proposed that the various epimerization patterns also depend on the concerted action of both the A- and the R-modules rather than only emanating from the catalytic activity of the A-modules [13]. The R-modules are suggested to modulate the interaction of the A-module with alginate and hereby affect the epimerization pattern as well as enhance the A-module activity. Also, all epimerases are Ca^2+^ dependent. Besides being important for the structural integrity of the protein, Ca^2+^ might also have other functional role(s) for the epimerase interaction with alginate [17].

The non-covalent interactions occurring between enzymes and alginate chains are comprised of hydrogen bonds, hydrophobic as well as electrostatic and van der Waals interactions [13]. In the case of epimerases (from a mutant library) yielding G-block in their product polysaccharides, recent studies carried out by Tøndervik and co-workers [13] showed that the binding groove on the A-module of AlgE6 possess more amino acid residues as compared to the other G-block producing epimerases that is promoting ionic bonding, hydrogen bonding and hydrophobic interactions (e.g. as mediated by Arg, Leu, Ser or Asn residues). These additional interactions are suggested to explain the ability of AlgE6 to form the longest G-stretches of all epimerases [13]. These results also illustrate how minor alterations in the epimerase composition can have significant consequences on the enzymatic activity and the final product. This effect was also neatly shown by substituting an electrically neutral tyrosine with a negatively charged aspartic acid residue in an AlgE4 A-module which naturally only produces alternating MG blocks. This might indicate that GG-formation takes place because the enzyme moves only one residue forward instead of two before making the next epimerization reaction, and hereafter the enzyme dissociates from the alginate polymer [13]. Naturally occurring epimerases, as well as their recombinant chimeric forms, are therefore powerful tools for preparation of alginates with particular residue sequences. The upgraded alginates thus obtained are characterized by enhanced functionality and bespoke physicochemical parameters, such as diffusion rates and mechanical properties in their hydrogel state. An increased understanding of the molecular interactions between epimerases and alginates will allow for precise control over the reaction and enhanced alginate modification possibilities.

Here, we characterize the nature of alginate-epimerase interactions by single-molecule techniques using both optical tweezers (OT) and atomic force microscopy (AFM). These techniques allow us to gain insight into the energy landscape of the complexes in questions and provide estimates for parameters such as lifetimes and free energies for transitions from a bound to an unbound free state. Such analysis is based on developed theoretical framework for interpretation of single-molecule force spectroscopy experiments established over the last decades [18–25]. These have previously been applied to characterize biomolecular interaction couples such as biotin-streptavidin [26], various receptor–ligand pairs [27–29], within DNA duplexes [30], mucin-mucin [31], oligonucleotide duplexes[30], DNA-LexA [32] and others systems, as e.g., reviewed [33]. AFM has also previously been applied to gain insight into alginate-epimerase interactions [34, 35] as well as the interaction between alginate and mucin [36, 37]. Compared to these previous studies, the combination of optical tweezers and AFM as applied in the present paper allow us to extend the accessible loading rate range investigated. Furthermore, the consequence of the modular composition of the epimerases was investigated by including the A-module of AlgE6 (AlgE6A), in addition to the enzymes AlgE4 and AlgE6 in the interaction studies.

## Materials and Methods

### Polysaccharides

Polymannuronic acid (poly-M) used in the present study was produced by an AlgG negative strain of *Pseudomonas fluorescens* NCIMB 10525 [38]. Poly-MG was made by *in vitro* epimerization of poly-M using AlgE4. Poly-M was dissolved in deionized water. Aqueous buffer (50 mM MOPS at pH 6.9, 2.5 mM CaCl_2_ and 10 mM NaCl) was added to the poly-M solution and preheated for one hour at 37°C. Hereafter, AlgE4 enzyme [14] isolated from *Hansenula polymorpha* (NOBIPOL and SINTEF, Trondheim, Norway) dissolved in deionized water was added to the poly-M solution at a final ratio of 1:150 epimerase: poly-M (w/w). The epimerization proceeded at 37°C for 48 hours under stirring before the reaction was terminated by adding a 50 mM EDTA solution. The solution with poly-MG was dialyzed against first 3 times 50mM NaCl and then 3 times MilliQ (MQ) water before freeze drying. The molecular weight was analyzed by size exclusion chromatography using a multi-angle light scattering detector. Samples were dissolved (1 mg/mL) in 0.15 M NaNO3/0.01 M EDTA (pH = 6) and injected (200 μL) into an HPLC system containing two serially connected columns (TSK G-6000+5000 PWXL) (Tosoh Bioscience LLC, PA, USA) connected to a Dawn DSP multi-angle laser light scattering photometer (λ = 633 nm).

The fraction of G-residues and diads of the monomer residues were determined to F_G_ = 0.47 in the epimerized alginate using mild acid hydrolysis and NMR spectroscopy as previously described [39]. The properties of the employed alginates are summarized in Table 1.

**Table 1:** Alginate and epimerase properties. The weight average molecular weight (M_w_), the polydispersity index determined as M_w_/M_n_ where M_n_ is the number average molecular weight and the fractions of α-L-GulA (G), F_G_, and diad fractions F_GG_ and F_GM_, where M denotes β-D-ManA, were determined for the alginate samples as described in the text. The unit of the epimerase activities (RU) is described in the text.

Alginates	M_w_	M_w_/M_n_	F_G_	F_GG_	F_GM_
Mannuronan	176 kD	1.8	0.00	0.00	0.00
Poly-MG			0.47	0.00	0.47
Epimerases	Activity				
AlgE4	1516	RU/mol			
AlgE6	971	RU/mol			
Alge6A	1280	RU/mol			

### Epimerases

Production and purification of AlgE4ds, AlgE6 and the A-module of AlgE6 were performed as previously described [40]. After purification the protein samples were dialyzed against 2mM HEPES pH 6.9 and 5 mM CaCl_2_, freeze-dried and stored at -20°C until used. The relative activities RU/mol of the enzymes were determined using a previously reported assay [13] where 1 RU is defined as increase in optical absorbance at 230 nm of 1 following 4 hours inclubation of the epimerized product with a G-lyase.

### Chemicals

The chemicals 1-(3-dimethylaminopropyl)-3-ethylcarbodiimide hydrochloride (EDAC), acetic acid, calcium chloride, hydrochloric acid, methanol, 2-methylpyridine borane complex solution, HEPES buffer, *N* ^1^-(3-Trimethoxysilylpropyl)diethylenetriamine (referred to as amino-silane in the following) were purchased from Sigma-Aldrich. N- (3-Trimethoxysilylpropyl)ethylenediamine triacetic acid trisodium salt (referred to as carboxyl-silane in the following) was purchased from ABCR GmbH & CO. KG. The amino-terminated beads (2.07 μm nominal diameter) and the carboxyl-terminated beads (3.21 μm nominal diameter) were obtained from Spherotech Inc. The deionized Milli-Q-water used had a resistivity of 18.2 MΩ cm (Milli-Q unit, Millipore).

### Preparation of samples for optical tweezers studies

**Fig 2:**
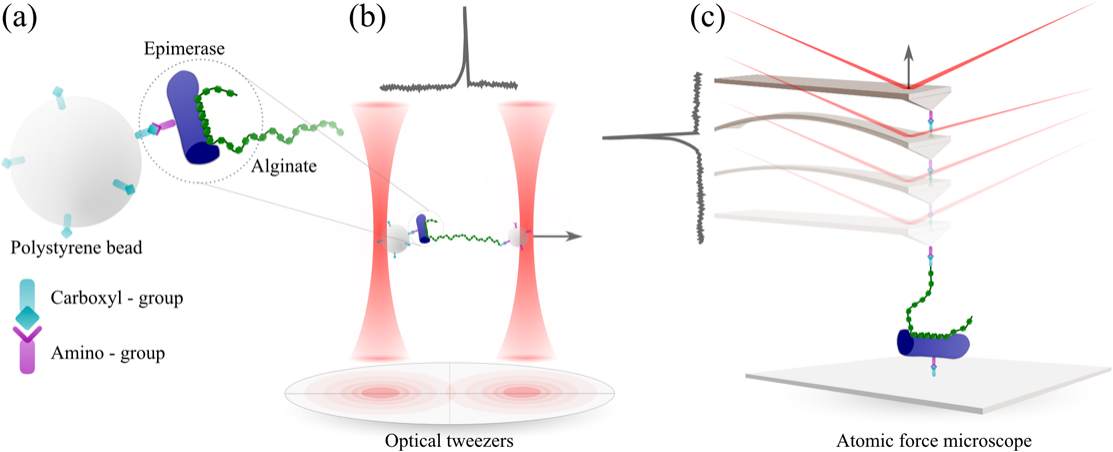
Schematic of the techniques used to study alginate-epimerase interactions. *(a)* Epimerase protein immobilized onto a polystyrene bead. The two force probes optical tweezers *(b)* and atomic force microscopy *(c)* display different force ranges associated to the difference in the spring constant of the optical traps and the AFM cantilever. Typical force-displacement curves displaying a force jump, as obtained when separating two functionalized and trapped polystyrene beads (b) or functionalized mica surface and AFM cantilever (c), are included. The force jumps reflect that the two surfaces were interconnected through an alginate-epimerase interaction, which was disrupted when increasing the separation distance between the two surfaces.

The alginates and epimerases were immobilized onto polystyrene beads terminated at their surface by amino- or carboxylic groups (Spherotech, Lake Forest, Illinois). The size of the beads was optimal for trapping using the dual-trap optical tweezers. The detection of the bead position relative to the laser beam was based on back focal plane interferometry, using 3.5 MHz bandwidth InGaAs photodiodes. The trap stiffness was determined for each trap prior to each experiment and varied in the range 5x10^-5^ to 1.5x10^-4^ N/m. All preparation steps for the polysaccharides and epimerases were carried out in room temperature (20°C). Poly-M and poly-MG were covalently anchored to amino-terminated beads (nominal diameter: 2.07 μm) utilizing EDAC (1 mg/ml in 50 mM boric acid buffer pH 5.8) to catalyze bond formation between amino groups on the beads and carboxyl groups on the alginate residues (Fig 2A) [41]. An incubation time in the range 2-3h combined with alginate concentrations in the range 0.1–0.2 mg/ml gave force curves in which signatures reflecting single molecule interaction with the epimerases present in about 10% of the retraction curves, ensuring a high probability for single molecular bonds [42].

The epimerases were anchored to carboxyl-terminated beads (nominal diameter: 3.21 μm) using EDAC (1 mg/ml in 50 mM boric acid buffer pH 5.8) to catalyze bond formation between amino groups on the epimerases and carboxyl groups on the beads (Fig 2A). The incubation time was 2-3h, and the epimerase concentrations were 0.1–0.3 mg/ml. The alginate—and epimerase functionalized beads were mixed and suspended in 50 mM HEPES with 10 mM CaCl_2_ at pH 6.9 for the experiments using the optical tweezers. The sample solution investigated using OT has a final bead concentration of about 1 x 10^6^ beads per ml for the beads with a diameter equal to 2.07 μm and about 3 x 10^5^ beads per ml for the beads with a diameter equal to 3.21 μm. The sample solution was placed between two glass slides and the sample cell was sealed using nail polish. Prior to all measurements, one bead of each type was identified based on their size difference visible in the microscope (2.07 μm or 3.2 μm) and captured. The trap stiffness of each trap was calibrated from power spectra obtained by tracking the 3D Brownian motion of the beads [43]. Dual-beam experiments were carried out with a bead contact time of 0.5-1s and approach/retraction-speeds in the range 0.5–4 μm/s.

### Preparation of samples for atomic force microscopy studies

The alginates (poly-M and poly-MG) were covalently anchored to AFM-tips (cantilevers from Veeco OTR4-10 Si_3_N_4,_ nominal spring constant 0.02 N/m) employing a previously described procedure [34, 35]. Briefly, the AFM-cantilevers were cleaned by immersing in a 1:1 v/v solution of MeOH/HCl for 30 min, rinsed in MQ -water, amino-silanized (1% (v/v) freshly prepared solution of amino-silane in 1 mM acetic acid) and rinsed in MQ -water. The alginates were covalently anchored to the functionalized AFM-tips through EDAC (1 mg/ml 50 mM boric acid at pH 5.8) catalyzed reactions between amino groups on the silane and carboxyl groups on the alginic acid residues (Fig 2C). The incubation time was 2–3 h and the concentrations of the alginates were 0.1 mg/ml.

Epimerases were covalently anchored to the mica slides using the following procedure. Mica slides were cleaved and immersed in a 1:1v/v solution of MeOH/HCl for 30 min, rinsed with MQ -water, carboxyl-silanized for 20 min (1% (v/v) freshly prepared solution of trimethoxysilylpropyl-triethylenetriamine in 1 mM acetic acid) and rinsed in MQ -water. The covalent bond formation between the silane on the mica slides and the epimerases were catalyzed by EDAC (1 mg/ml 50 mM boric acid pH 5.8). The incubation time was 2-3h and the epimerase concentrations were 0.1–0.3 mg/ml. These experimental conditions gave AFM force–distance curves revealing signatures of forced rupture of alginate–epimerase complexes in around 10% of the approach/retract cycles. All preparation steps for the polysaccharides and epimerases were carried out in room temperature (20°C).

All epimerase functionalized mica slides and alginate functionalized cantilevers were freshly prepared for each experiment and were neither dried nor stored. The procedure employed for the covalent attachment of the epimerases have previously been reported to yield active enzymes immobilized to the surface with one active enzyme per 24 nm^2^ of the surface [34]. The alginate functionalized cantilevers were immersed in a liquid cell containing 50mM HEPES buffer and 10mM CaCl_2_ at pH 6.9 with the epimerase functionalized mica slide attached to the bottom of the cell. Prior to all measurements, the cantilever spring constant was calibrated by measurements of the resonance frequency of the cantilever away from the mica slide using the thermal tune principle [44]. The deflection sensitivity calibration was carried out by pressing the cantilever towards the mica surface. The measurements were carried out by approaching the alginate-functionalized tip towards the epimerase-functionalized mica slide. Once in contact with the surface, a waiting time of 0.5-1s was used before retraction along the z-direction with speeds in the range 0.5–4 μm/s.

### Dynamic force spectroscopy

The optical tweezers (JPK NanoTracker) and the force robot (JPK ForceRobot 300); an atomic force microscope dedicated to translations in the z-direction, were utilized to investigate single-molecule interactions between poly-M and poly-MG alginates with the epimerases AlgE4, AlgE6 and the A-module from AlgE6 (Figs 1 and 2). Due to differences in the trap stiffness of the optical tweezers (nominal value of ~100 pN/μm depending on bead size) and the cantilever spring constant on the force robot (nominal value 20 nN/μm), forces between 0.5–100 pN were detectable with the OT, while the AFM supported force measurements beyond 5 pN.

The experimentally determined energy landscapes of the macromolecular interactions were interpreted based on developed theoretical framework [18–25] as outlined in the following. The interpretation of the single-molecule force spectroscopy experiments here focuses on interaction strengths and lifetimes being the key parameters. The dissociation rate related to the transition from a bound to an unbound free state is dependent on the applied force as:

$$k(f)=k^{0}{\left(1-\frac{\nu fx_{\beta }}{\Delta G^{\#}}\right)}^{\frac{1}{\nu -1}}\text{exp}\left\{\beta \Delta G^{\#}\left[1-{\left(1-\frac{\nu fx_{\beta }}{\Delta G^{\#}}\right)}^{\frac{1}{\nu }}\right]\right\}$$ (1)

where *k* ^0^ is the dissociation rate at zero force, *x*_*β*_ is the distance to the transition state in the energy landscape along the reaction coordinate, *f* is the applied force, *β* is the inverse of the thermal energy and Δ*G* ^#^ is the free energy of activation in absence of force. The external perturbation tilts the energy landscape about the reaction coordinate, yielding an increased likelihood of dissociation. Moreover, assuming that the quasi adiabatic criteria holds i.e. the dissociation rate is much slower than the characteristic relaxation time in the bound state [21, 23], the distribution of unbinding forces at constant retraction-speeds as e.g. extracted from a force ramping experiment, is given as:

$$P(f)=\frac{1}{r_{f}}k(f)\text{exp}\left\{\frac{k^{0}}{\beta x_{\beta }r_{f}}\right\}\text{exp}\left\{-\frac{k(f)}{\beta x_{\beta }r_{f}}{\left(1-\frac{\nu fx_{\beta }}{\Delta G^{\#}}\right)}^{1-\frac{1}{\nu }}\right\}$$ (2)

Where *r*_*f*_ = *df* / *dt* = *V k* (*k* is the spring constant of the system) defined as the loading rate and is a controllable parameter through the retraction-speed *V* set by the operator. The phenomenological theory [22, 45] is restored for *v* = 1, yielding a liner increase in the most probable unbinding force f* with ln (*r*_*f*_). In this interpretation, the distance to the transition state *x*_*β*_ is independent of the applied force as a consequence of the assumption that the wells and peaks in the energy landscape are sharp and harmonic. Furthermore, the cusp-barrier/well and the linear-cubic free energy surface is attained for *ν* = 1/2 and *ν* = 2/3, respectively. Asymptotic expressions for the mean rupture force and force variance are given as

$$\langle f\rangle ≅\frac{\Delta G^{\#}}{\nu x_{\beta }}\left\{1-{\left[\frac{1}{\beta \Delta G^{\#}}\text{ln}\left(\frac{k^{0}\;\text{exp}\left(\beta \Delta G^{\#}+\gamma \right)}{\beta x_{\beta }r_{f}}\right)\right]}^{\nu }\right\}$$ (3)

$$\sigma_{f}^{2}≅\frac{\pi^{2}}{6{\left(\beta x_{\beta }\right)}^{2}}{\left[\frac{1}{\beta \Delta G^{\#}}\text{ln}\left(\frac{k^{0}\;\text{exp}\left(\beta \Delta G^{\#}+\gamma \right)}{\beta x_{\beta }r_{f}}\right)\right]}^{2\nu -2}$$ (4)

where *γ* ≈ 0.577 is the Euler-Macheroni constant. Parameter <f> is found to be good estimate of the maximum of the rupture-force distribution f* when γ is set to zero. The predicted constant force variance (Eq 4) emerges from the assumption of *x*_*β*_ being independent of the applied force. Observations of a force dependent variance therefore indicate that an underlying assumption of the phenomenological approach is not fulfilled. Models accounting for changes in the transition state locations for high force regions e.g. cusp- or the linear cubic theory are in these cases needed. Additionally, if the quasi-adiabatic assumption is valid, constant retraction-speed experiments (measuring *P(f)*) and constant force experiments (measuring *k(f)*) yield the relationship [18, 19]:

$$k(f)=\frac{r_{f}P(f)}{1-\int_{0}^{f}P(f^{'})df^{'}}\forall r_{f}$$ (5)

This relationship between the constant-speed rupture-force analysis and the constant-force rupture-rate can thus be used to validate the quasi-adiabatic assumption. Practically, this is done by converting data of the individual bins from all constant-speed rupture-force histograms into corresponding constant-force rupture-rates using:

$$k(f)=\sum_{n=1}^{N_{h}}\sum_{p=1}^{N_{b}}k(f_{1n}+(p-1/2)\Delta f_{n})=\sum_{n=1}^{N_{h}}\sum_{p=1}^{N_{b}}\left[\;\frac{h_{pn}{\langle r_{f}\rangle }_{n}}{\left(\frac{h_{pn}}{2}+\sum_{i=p+1}^{N_{b}}h_{in}\right)\Delta f_{n}}\right]$$ (6)

In Eq 6, *N*_*h*_ is the total number of histograms, *N*_*b*_ is the total number of bins for the governing histogram, *f*_*1n*_ is the force magnitude of the first histogram bin for histogram *n*, Δ*f*_*n*_ is the bin-width of histogram *n*, *h*_*pn*_ is the height of bin *p* for histogram *n* and 〈*r*_*f*_〉_*n*_ is the mean loading rate within histogram *n*.

### Analytical routines

**Fig 3:**
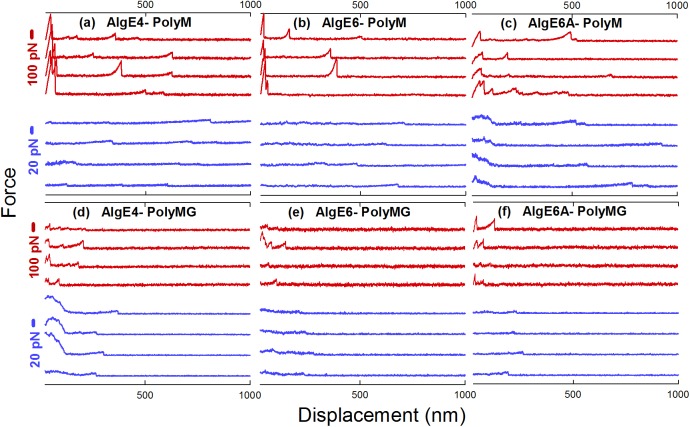
Gallery of rupture events of the various AlgE-poly-M *(a*,*b*,*c)* and AlgE-poly-MG *(d*,*e*,*f)* molecular pairs. The red curves are recorded with AFM, while the blue curves represent forced ruptures recorded with OT. The displacement scale corresponds to the z-piezo translation distance, and bead separation for the data collected employing AFM and OT, respectively. Some of the AFM curves display interactions at short displacement distances (~ 0–50 nm) that may reflect non-specific interaction between the AFM-tip and the mica slide (e.g. red curves in a,b, and c). Bead-bead interactions are in some recordings present at the adhesion region of the OT experiments, such as the ones in *(c)* and *(d)*. Interactions in the adhesion region either due to AFM-tip mica slide contact in the AFM experiments or due to strong bead-bead interactions in the OT were not included in the analysis as single-bond rupture events.

**Fig 4:**
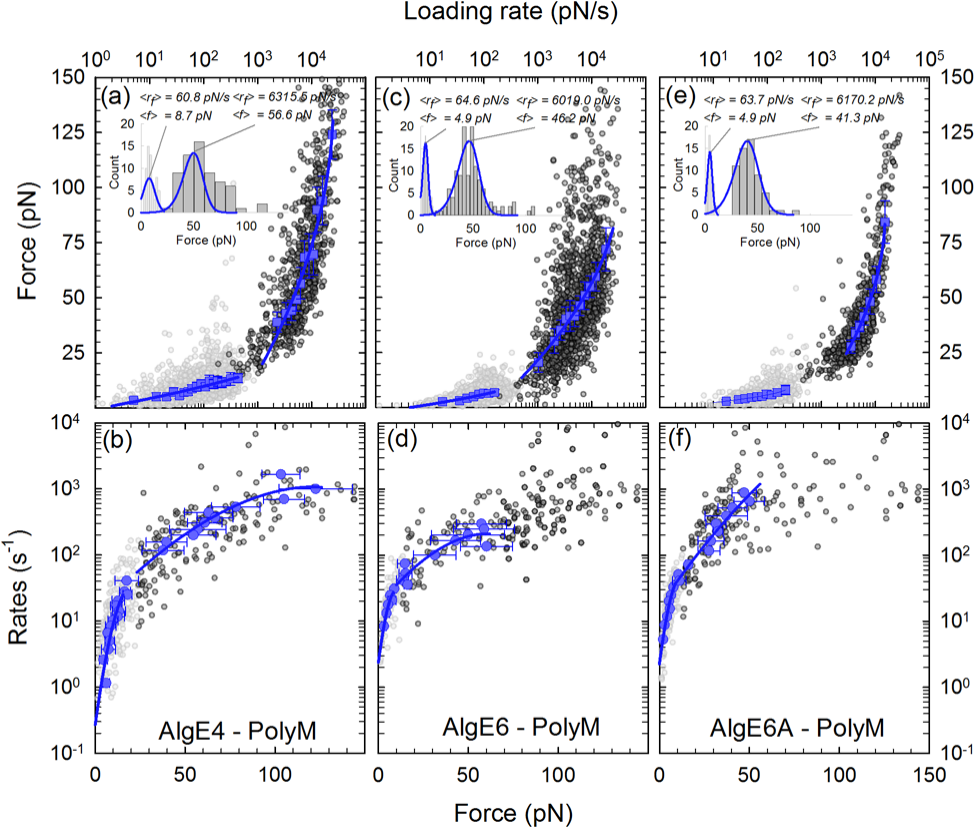
Interactions of AlgE–poly-M complexes determined by direct unbinding. *(a*, *c*, *e)* Constant-speed rupture-force representation of the interactions i.e. mean forces versus mean loading rates (symbols) and fits of Eq 3 using *ν* = 1/2 (lines) to the experimental data. The estimates of the molecular interaction parameters are shown in Table 2. The light grey data points are forced ruptures recorded with the OT, while the dark grey data points are forced ruptures recorded with the AFM. By combining the two techniques we can access a larger range of loading rates as can be seen in *(a*, *c*, *e)*. The inserts show two selected histograms of the unbinding forces, within the low and high loading rate regions, respectively, indicate typical distributions of the data within the loading rate intervals. The distribution of unbinding force P(f) (Eq 2) for *ν* = 1/2 (lines) is included on top of the histograms. *(b*, *d*, *f)* Constant-force rupture-rate representation with fits using *ν* = 1/2 of the interactions for the poly-M-complexes. The data are presented as mean rates versus mean forces obtained analytically from the constant-speed rupture-force experiments (Eq 6). The resulting estimates of the molecular interaction parameters are shown in Table 3.

**Fig 5:**
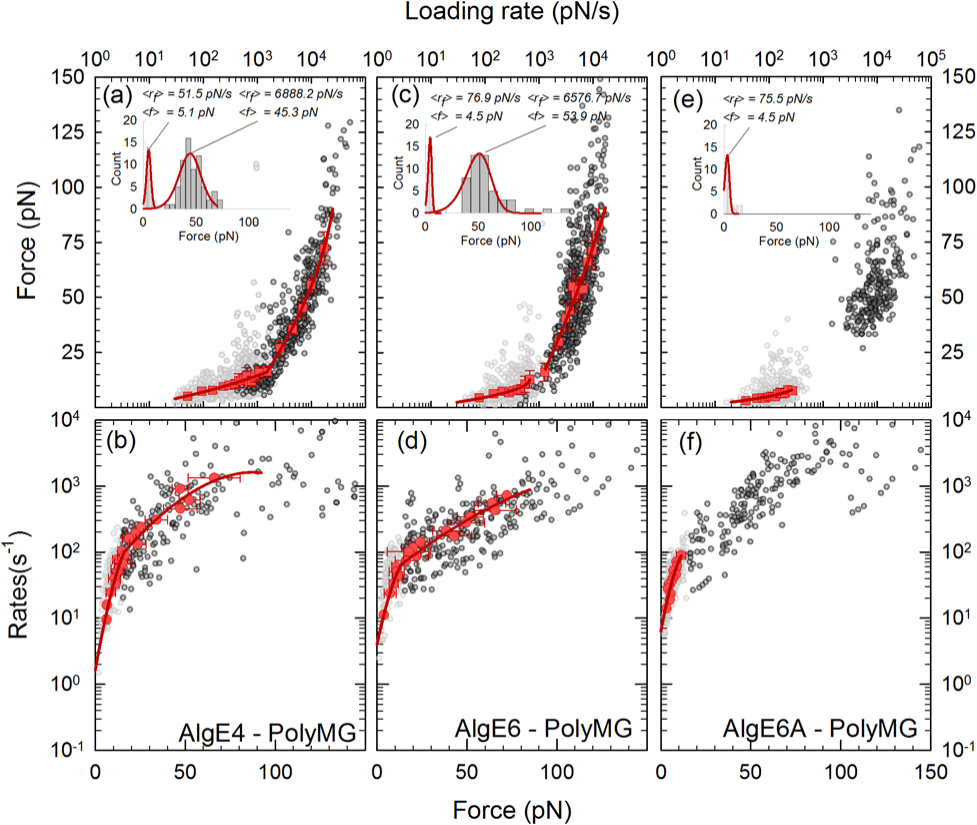
Interactions of AlgE–poly-MG complexes determined by direct unbinding. *(a*, *c*, *e)* Constant-speed rupture-force representation of the interactions i.e. mean forces versus mean loading rates (symbols) and fits of Eq 3 with *ν* = 1/2(lines) to the experimental data. The estimates of the molecular interaction parameters are shown in Table 2. The light grey data points are forced ruptures recorded with the OT, while the dark grey data points are forced ruptures recorded with the AFM. By combining the two techniques we can access a larger range of loading rates as can be seen in *(a*, *c*, *e)*. The inserts show two selected histograms of the unbinding forces, within the low and high loading rate regions, respectively. The histogram plots, one for each energy barrier, exhibit the distribution of unbinding forces for which Eq 2 with *ν* = 1/2(lines) was fitted. *(b*, *d*, *f)* Constant-force rupture-rate representation with *ν* = 1/2 of the interactions for the poly-M-complexes. The data are presented as mean rates versus mean forces obtained analytically from the constant-speed rupture-force experiments (Eq 6). The resulting estimates of the molecular interaction parameters are shown in Table 3.

The underlying stochastic nature of non-covalent molecular pair-interactions dictates a large number of observations to yield robust estimates of parameters of the energy landscape. Force- displacement curves, i.e evidence of molecular interactions (Fig 3), were collected for all the various interaction pairs of alginates and epimerases. The force magnitudes and the corresponding loading rates were extracted from each curve by determining the magnitude and slope of all force jumps, respectively. The employed procedure using a linear approximation of the increase in force just prior to the unbinding event conform to *r*_*f*_ the fit of a wormlike chain model with persistence length 10 nm for the alginates [46, 47] within 5% and 10% for unbinding forces up to 20 and 55 pN, respectively. This is considered adequate within the noise level of the data. The acquired force magnitudes with corresponding loading rates for each interaction couple were accumulated to generate force v.s. ln(loading rate) plots (Fig 4A, 4C and 4E and Fig 5A, 5C and 5E). Moreover, difficulties in separating single- from multiple interactions in individual force-displacement curves can in addition to lack of data complicate the analysis. Possible effects of multiple interactions were reduced by using the mean forces (Eq 3) of the interquartile range for given force loading rate intervals. This is an alternative to generating constant-speed rupture-force histograms of the force loading rate intervals and estimating most probable unbinding forces from fits of *P(f)* to individual histograms, which requires large datasets to yield robust estimates. The adaption of mean forces applies since an approximation of the true distributions to a Gaussian distribution is valid near the mean such that the mean force can be assumed to lie near the most probable unbinding force [19]. To obtain estimates of *x*_*β*_, *τ* ^*0*^ and *ΔG* ^*#*^ for given values of *v*, master curves were generated by fits of Eq 3 to the mean forces with corresponding mean loading rates. Furthermore, rupture-force histograms were generated for each force loading rate interval and re-plotted as *k(f)* using Eq 6, again yielding estimates of *x*_*β*_, *τ* ^*0*^ and *ΔG* ^*#*^. This was performed as a consistency test and to validate the quasi-adiabatic assumption. Consistency between the estimates obtained from the two methods is thus a necessity. Each dataset was analyzed by dividing the range of force-loading rates into an interval with mean loading rates with corresponding mean forces, from which *x*_*β*_, *τ* ^0^ and Δ*G* ^#^ were determined by fits of Eq 3. However, as these estimates depend slightly on the selection of the intervals, parameters *x*_*β*_, *τ* ^0^ and Δ*G* ^#^ were estimated for various selection of intervals. The number of datapoints in each interval was kept at a minimum of one hundred to provide a sufficient basis for histograms to be used to fits of Eq 2. The mean of the parameters, 〈*x*_*β*_〉, 〈*τ* ^0^〉 and 〈Δ*G* ^#^〉, were then calculated based on all collected *x*_*β*_, *τ* ^0^ and Δ*G* ^#^ for that dataset. Over-fitting was accounted for by calculating predicted coefficient of determination $R_{\text{Pred}}^{2}$ values from which all regressions yielded similar coefficient of determination, *R* ^2^, and $R_{\text{Pred}}^{2}$ in the range 0.85–0.95.

## Results and Discussion

### Energy landscape parameters

The force-displacement curves recorded with the OT and the AFM revealed unbinding events at separations typically in the range 50 –up to 500 nm and even beyond that for the poly-M—epimerases, and mainly in the interval 50–200 nm for the poly-MG–epimerases (examples are shown in Fig 3). The clearly identifiable unbinding events were preceded with an increase in force reflecting a stretching of the alginate chains (Fig 3). Such force distance profiles, at difference with patterns with extensive saw-tooths or plateaus reported for other cases [48–50], indicate interactions between alginate chains and epimerase enzymes forming enzyme-substrate complexes that have sufficient lifetime to yield the signature of polymer stretching. These complexes are formed by alginates bound to the active site of the enzyme, as determined by previously reported inhibition test [34]. Based on the collected force traces, we estimate the noise level to be ~5pN for the AFM and ~0.5pN for the OT. Additional control measurements were performed by allowing the alginates poly-M and poly-MG to interact with carboxyl-silanized surfaces, or by allowing the three epimerases to interact with amino-silanized surfaces were carried out to examine the specificity of the observed alginate-epimerase interactions. The finding that only 1% of the of force-displacement curves displayed unbinding features and if present, these were all present in the adhesion region (i.e. the contact point between the polystyrene beads used in the optical tweezers experiments, or the cantilever tip with the mica slide for the OT and AFM experiments, respectively), indicate no contamination of unspecific interactions in the data included in the analysis. Interactions in the adhesion region, if present as indicated in some of the force-distance profiles in Fig 3, were not included in the data extracted to assess the specific molecular interactions.

The combined analysis of AFM and OT data reveal that all six complexes investigated displayed two slopes in the f – ln(r_f_) as shown for the poly-M and poly-MG complexes in Fig 4A, 4C and 4E and Fig 5A, 5C and 5E. In the case of the AlgE6A –poly-MG interaction the number of datapoints was insufficient to yield reliable parameter estimates (Table 2). For the other systems, the observations suggest the existence of an inner and an outer energy barrier each characterized by its *x*_*β*_ value (Table 2).

**Table 2:** Averages of energy landscape parameters for epimerase–alginate interactions. The parameters *x*_*β*_, *τ* ^*0*^ and *ΔG* ^*#*^ were estimated using the constant-speed rupture-force analysis after multiple regressions of Eq 3 with *v* = 1/2 to observed mean forces for all force loading rate intervals. The number of force loading rate intervals was varied from 20 and up to 30 yielding 20–30 estimates of the most probable unbinding force at the corresponding mean loading rates. The number of mean force estimates was about the same for the inner and outer barriers. The resulting master curves for the poly-M and the poly-MG complexes are shown in Fig 4A, 4C and 4E and Fig 5A, 5C and 5E, respectively.

*Constant-speed rupture-force*							
		*Inner Barrier*			*Outer Barrier*		
		〈*x*_*β*_〉 *(nm)*	〈*τ* ^0^〉 *(s)*	*〈ΔG* ^*#*^ *〉 (k*_*B*_ *T)*	〈*x*_*β*_〉 *(nm)*	〈*τ* ^0^〉 *(s)*	〈*ΔG* ^*#*^〉 *(k*_*B*_ *T)*
	AlgE4	0.20±0.01	0.05±0.00	3.6±0.1	2.2±0.4	1.7±0.6	6.8±1.6
Poly-M	AlgE6	0.40±0.03	0.10±0.01	5.1±0.1	2.3±0.2	0.4±0.01	5.9±2.1
	AlgE6A	0.20±0.02	0.03±0.00	2.6±0.1	3.5±0.3	0.7±0.1	4.8±0.2
	AlgE4	0.30±0.01	0.03±0.00	3.5±0.1	1.7±0.2	0.4±0.05	8.0±1.9
Poly-MG	AlgE6	0.20±0.02	0.04±0.00	4.8±2.6	2.7±0.3	0.3±0.04	4.1±0.2
	AlgE6A	Insufficient data			2.8±0.2	0.3±0.01	3.8±0.1

Fits of Eq 2 to individual histograms (Fig 4A, 4C and 4E and Fig 5A, 5C and 5E) also indicated the existence of two barriers in the energy landscape of the interaction, where a distinct jump in *x*_*β*_, τ^0^ and ΔG^#^ for individual force loading rate intervals were evident in the intersection between the two lines for a given loading rate (not shown). These estimates were, however, less reliable than those obtained from the master curves due to limited number of data for individual force loading rate intervals.

The estimates of the parameters of the energy landscapes obtained using the constant-speed rupture-force and constant-force rupture-rate approach were in good agreement (Tables 2 and 3). This was deduced by converting the histograms underpinning the f – ln(r_f_) representations (Fig 4A, 4C and 4E and Fig 5A, 5C and 5E) to ln(k) − f relations (Eq 6) for each of the poly-M and poly-MG interacting with the three epimerases (Figs 4B, 4D, 4F, 5B, 5D and 5F). This finding indicates that the quasi-adiabatic assumption holds for the range of loading rates investigated in this study.

**Table 3:** Average values of energy landscape parameters obtained for the constant-force rupture-rate analysis. The estimates were obtained using multiple regressions of Eq 1 (*v* = 1/2) after conversion to constant-force rupture-rate (Eq 6) to mean rates with corresponding mean forces. The number of force loading rate intervals employed and procedure are as described in Table 2. The resulting master curves for the poly-M and the poly-MG complexes are shown in Figs 4B, 4D, 4F, 5B, 5D and 5F, respectively.

*Constant-force rupture-rate*							
		*Inner Barrier*			*Outer Barrier*		
		〈*x*_*β*_〉 *(nm)*	〈*τ* ^0^〉 *(s)*	*〈ΔG* ^*#*^ *〉 (k*_*B*_ *T)*	〈*x*_*β*_〉 *(nm)*	〈*τ* ^0^〉 *(s)*	*〈ΔG* ^*#*^ *〉 (k*_*B*_ *T)*
	AlgE4	0.30±0.03	0.09±0.03	6.6±0.3	1.9±0.3	2.5±0.8	6.8±0.2
Poly-M -	AlgE6	0.30±0.10	0.10±0.06	7.4±2.4	3.0±0.5	0.8±0.3	5.9±0.5
	AlgE6A	0.50±0.10	0.10±0.07	6.0±0.9	2.7±0.6	0.7±0.2	9.0±1.1
	AlgE4	0.35±0.03	0.03±0.01	6.9±0.6	1.8±0.2	0.6±0.2	7.3±0.7
Poly-MG	AlgE6	0.30±0.02	0.03±0.00	6.0±0.8	1.5±0.7	0.2±0.1	5.9±1.0
	AlgE6A	Insufficient data			2.5±0.4	0.3±0.1	8.3±2.9

**Fig 6:**
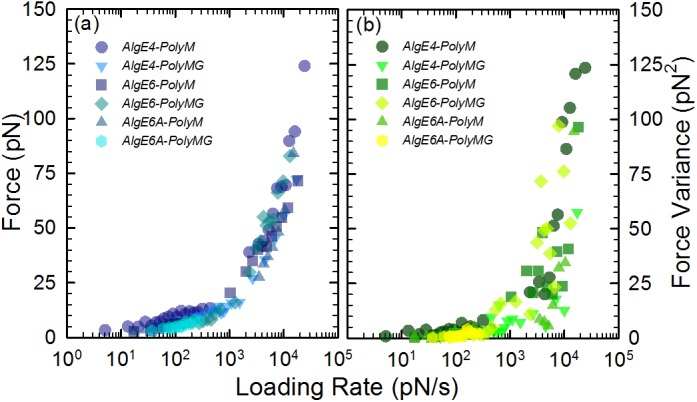
Comparison of mean force (a) and force variance (b) versus force loading rate for the alginate-epimerase pairwise interactions.

The fit of the phenomenological theory (ν = 1) (Eqs 1 or 3) to the constant-force mean rupture-rates and constant-speed mean rupture-forces data gave deviations from the expected trends, in particular for the higher loading rates. This can be interpreted as shifts of the transition states along the reaction coordinate as the energy landscape is tilted i.e. *x*_*β*_ becomes force dependent and decreases with increasing force. In such cases, the variance of the force, *σ*_*f*_ (Eq 4) is not constant for given *x*_*β*_, but rather, increases with force (increasing error bars towards higher loading rates in Figs 4A, 4C, 4E, 5A, 5C, 5E and 6). In contrast, good fits were obtained both for *ν* = 1/2 and *ν* = 2/3 i. e. the cusp-barrier/well and the linear-cubic free energy surface assumptions, respectively. In the present study, identification of the shape of the energy landscape is not required given the similarities in the estimates for both the cusp-barrier/well and the linear-cubic free energy surfaces. The further analysis was performed based on the parameters obtained from the constant-speed rupture-force analysis for *ν* = 1/2.

### Epimerase interactions with poly-M and poly-MG

The locations of energy barriers for the AlgE4- and AlgE6-poly-M complexes determined in the present study using AFM yields an estimate of *x*_*β*_ in the range 0.2–0.5 nm (Tables 2 and 3, Inner barrier). These estimates are in the same range with that previously reported of 0.24nm for epimerase–alginate interactions [34, 35]. This range is similar to *x*_*β*_ of 0.35 nm reported for GAG self-interactions [48] and linear tetramannose interacting with a monoclonal antibody [51] both being examples being interactions involving carbohydrates. In the present study, we additionally identify another energy barrier with reaction length *x*_*β*_ in the range 1.5–3.5 nm (Tables 2 and 3), which is larger than that previously determined. These barriers were evident from the data obtained in the low force region accessible by optical tweezers. The presence of a second energy barrier located outside of the innermost barrier was in the present study evident in all complexes investigated. The value of the particular outer barrier *x*_*β*_ in the range 1.5–3.5 nm, compares with the values for the outer barrier of 1.2 nm reported for the P-selectin glycoprotein ligand interacting with L-selectin [20], *x*_*β*_ = 1.1 nm and 4.5 nm for homotypic and heteromeric interactions between extracellular domain constructs of E-cadherin [52] and *x*_*β*_ exceeding 2 nm for dissociation of dsDNA oligomers with number of basepairs larger than 20 [30]. It is also interesting to note that the outer barrier identified in the present study indicate a separation distance for dissociation that compares with an extended length of an oligosaccharide interacting with the epimerases in subsite model. In particular, amino acids involved in the alginate interactions at 6 subsites of the A-module of the AlgE4 epimerase have been identified [5], which correspond to binding of an alginate sequence of 6 consecutive monosaccharides with a length of about 3 nm, employing an average monosaccharide residue length of 0.5 nm. Thus, the observed outer barrier (Tables 2 and 3) indicate that a separation similar to the length of interacting oligosaccharide sequence with the epimerization enzyme is needed for the dissociation. In specific interactions between biological macromolecules characterized by more than one energy barrier, the inner energy barriers are rate-limiting when perturbed by smaller forces. Identification of the outer barriers and their parameter values will therefore advance the molecular understanding of the epimerase-polymer substrate/product interactions.

The fraction of force-distance curves showing interactions were less frequent for the poly-MG–epimerases than for the poly-M epimerases, despite of identical concentration of the poly-M and poly-MG used in the conjugation step. Moreover, force-distance curves of poly-MG–epimerase displayed unbinding events at shorter distances from contact (100 – 200nm), than for the poly-M–epimerases. These experimental challenges explain the difference in the number of observations included in the datasets between the poly-M and poly-MG complexes (1200–2600 anchoring events for the poly-M–epimerases compared to 700–1370 for the poly-MG- epimerase complexes). These differences were also observed in previous studies [34, 35], where it was suggested to arise from differences in chain flexibility between these alginate polymers. The poly-MG chains can be viewed to adopt a less extended state due to smaller persistence length and thus reducing the possible interaction distance.

**Fig 7:**
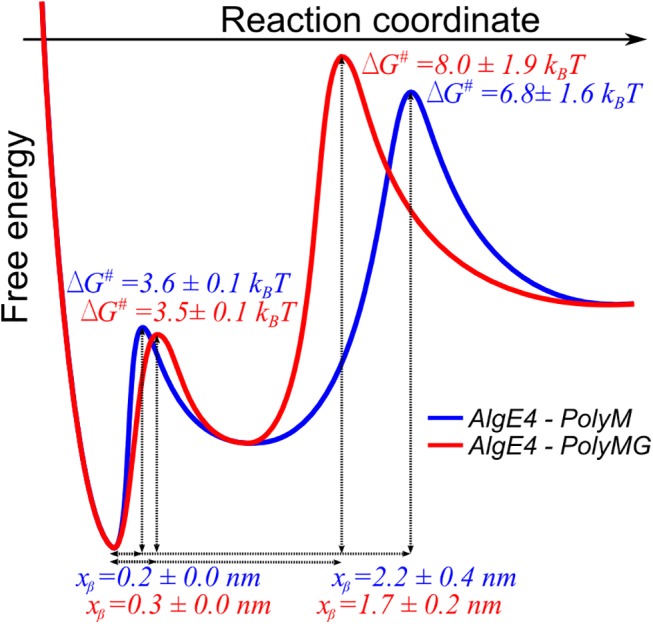
Schematic illustration of reconstructed energy landscapes for the AlgE4-poly-M and AlgE4 –poly-MG pairwise interactions based on the obtained parameters (Table 1).

The location of the barrier and the free energies of the interactions were for AlgE4 found to be similar for the two types of alginate substrates studied (Tables 2 and 3). Fig 7 present a schematics of the reconstructed energy landscapes for these pairwise interactions. It should be noted that estimates of the apparent activation energy for formation of the pairwise interactions are not obtainable based on the current analysis. The energy landscape illustrate, with the uncertainties as indicated, the pathway for the (forced) dissociation from the bound state over the outer barrier (low range of force loading rate) and inner barrier (higher range of force loading rate) representing the rate limiting step of the process.

Similar energy landscape parameters were also observed for the AlgE6- and AlgE6A-complexes, although the x_β_ values were slightly increased whereas the *∆G* ^*#*^ were decreased compared to complexes with AlgE4. It is interesting to compare the observed energies and reaction lengths with that of processive molecular systems. The energy barriers identified in the alginate–epimerase case (Tables 2 and 3) are all characterized by a free energy being less than the 20 k_B_T associated with hydrolysis of ATP (per molecule). The free energies determined in the present study are also below the 12 k_B_T work performed by the ATP catalyzed processive movement of kinesin along microtubule [53]. The recently reported 9 k_B_T energy difference between the local minima in a two-state model of the cell surface sulfatase Sulf1 catalyzing desulfation of glucosaminoglycans in a processive way [54] are comparable to the upper boundary of *ΔG* ^*#*^ determined for the epimerase–alginate interactions. The reaction lengths *x*_*β*_ found for the outer barriers are considered consistent with the accepted model of sub-site binding of the alginate to the epimerases, which for some complexes include up to 10 sugar residues [15].

The free energies of dissociation up to 8 k_B_T for the outer barrier in the epimerase-alginate case, and the smaller values for the inner barriers (Tables 2 and 3), indicate that thermal energy is sufficient to induce significant perturbations along the reaction pathway. The enzyme and substrate are thus not tightly locked at all times but are able to relocate. The different affinities determined for AlgE4-poly-M and AlgE4-poly-MG combined with the knowledge that the enzymes interact with the alginate polymer through electrostatic and hydrophilic forces (charged and polar amino acids) [13] can be considered essential in providing an energy landscape supporting the processive mode of action of these epimerases. The similarity of the free energies determined here, and that for the Sulf1 interacting with GAGs, furthermore suggest that epimerase–alginate free energies are in a range compatible with processive mode. This affirmation is based on the previously suggested inchworm movement of Sul1 on the GAGs [54]. The different affinities mediated by the various amino acids will also contribute to directing the orientation of the alginate in the epimerase binding groove [40]. After the epimerization of a sugar unit, an electrostatic switch between attraction and repulsion of the alginate chain by the enzyme is expected to occur, enabling propagation in a distinct direction.

Our results confirm that AlgE4 binds to poly-MG, which is a prerequisite of processivity. However, the lifetime of this interactions (0.4 ± 0.05s) is found to be shorter than the AlgE4- poly-M interaction (1.7 ± 0.6s). Although the lifetime differences were less pronounced the same trend was observed also for the other enzymes (Table 2). A lifetime of ~0.4 s without performing any further epimerization may indicate that the enzyme binds and stalls on the same position or that it moves along the chain to search for un-epimerized units.

It is interesting to compare the lifetimes of the complexes to the catalytic constants, i.e., the maximum number of substrate (M)- to product (G) monomer conversion per substrate per unit time, previously determined for these enzyme-substrate systems. A catalytic constant of 14s^-1^ (t_cat_ = 0.07s) has been measured at 37°C for AlgE4 [14]. Combining this information with the lifetime determined for AlgE4-poly-M complex (Table 2), leads to an estimate of ~12 sugar residues epimerized per productive binding. These findings agree with previous studies of the processive nature of AlgE4, where the AlgE4 was suggested to epimerize ~10 sugar residues per productive binding [15]. Proper account of a possible temperature dependence on the parameters should provide a more precise estimate of the degree of processivity compared to the present one, where a catalytic constant measured at 37°C is combined with unbinding data obtained at room temperature.

The similarity of the energy landscape estimates between AlgE6A and AlgE6 with both poly-M and poly-MG (Table 2) indicate that the A-module of AlgE6 determines the AlgE6-alginate binding strength, with little or no contribution from the R-module. These observations are in accordance with recently published data showing that the R-modules (R1R2R3) of AlgE6 in the absence of the A-module show only weak interaction to poly-M [40]. The same study reported strong interactions between the R-module of AlgE4 and poly-M. This is in accordance with the long lifetime of this complex relative to the other complexes studied (Table 2). The weak binding nature of the R-modules of AlgE6, their hierarchical compact structure and their multiple binding sites for Ca^2+^, is believed to play a crucial role in temporarily avoiding gelling of the alginate chain while the enzyme is still attached to the alginate. AlgE4, on the other hand, is in no need of this type of regulation since poly-MG only form very weak gels, and this might explain the documented alginate binding properties of the AlgE4 R module [2, 10, 11].

## Conclusion

We have used the force probes optical tweezers and atomic force microscopy to address he nature of alginate-epimerase interactions. The consequence of the modular composition of the epimerases was investigated by studying the interaction between alginate and the A-module of AlgE6 (AlgE6A), in addition to the naturally occurring epimerases AlgE4 and AlgE6. Through the use of optical tweezers in addition to AFM we gained access to a loading rate interval that is significantly wider than the interval accessible with only one of these probes. This combination of force probes thus provide a strong experimental basis for the conclusions outlined in the following. Our results support the previously hypotesized processive mode of action for AlgE4. Furthermore, our results reveal the existence of an additional energy barrier with reaction length larger than that previously determined. The data indicate a that thelifetime of the interactions between the studied epimerases and polymannuronic (poly-M) are significantly longer than the lifetimes between these epimerases and the polyalternating (poly-MG) alginates. This tendency is particularly pronounced for AlgE4, a poly-MG forming epimerase, and less pronounced for AlgE6, a G-block forming epimerase that thus also accepts poly-MG alginate as a substrate. The free energy of the interactions was determined to be in the range 3.8 to 9 k_B_T for the outer rate limiting energy barrier of these interactions. Thermal energy is thus sufficient to induce significant perturbations along the reaction pathways, and as a consequence the enzymes are not tightly locked to the substrate at all times but are able to relocate. Together with the different affinities determined for AlgE4-poly-M and AlgE4-poly-MG, these relatively low free energies are essential for providing an energy landscape supporting a processive mode of action of these epimerases.
